# Jedi-1 deficiency increases sensory neuron excitability through a non-cell autonomous mechanism

**DOI:** 10.1038/s41598-020-57971-2

**Published:** 2020-01-28

**Authors:** Alexandra J. Trevisan, Mary Beth Bauer, Rebecca L. Brindley, Kevin P. M. Currie, Bruce D. Carter

**Affiliations:** 1 Department of Biochemistry, Vanderbilt University School of Medicine, Nashville, TN USA; 2 Anesthesiology, Vanderbilt University School of Medicine, Nashville, TN USA; 3 Department of Biomedical Sciences, Cooper Medical School of Rowan University, Camden, NJ USA; 4 Vanderbilt Brain Institute, Vanderbilt University School of Medicine, Nashville, TN USA

**Keywords:** Developmental biology, Differentiation, Cell death in the nervous system, Molecular neuroscience, Sensory processing, Somatosensory system, Pain, Neuroscience, Glial biology, Schwann cell

## Abstract

The dorsal root ganglia (DRG) house the primary afferent neurons responsible for somatosensation, including pain. We previously identified Jedi-1 (PEAR1/MEGF12) as a phagocytic receptor expressed by satellite glia in the DRG involved in clearing apoptotic neurons during development. Here, we further investigated the function of this receptor *in vivo* using Jedi-1 null mice. In addition to satellite glia, we found Jedi-1 expression in perineurial glia and endothelial cells, but not in sensory neurons. We did not detect any morphological or functional changes in the glial cells or vasculature of Jedi-1 knockout mice. Surprisingly, we did observe changes in DRG neuron activity. In neurons from Jedi-1 knockout (KO) mice, there was an increase in the fraction of capsaicin-sensitive cells relative to wild type (WT) controls. Patch-clamp electrophysiology revealed an increase in excitability, with a shift from phasic to tonic action potential firing patterns in KO neurons. We also found alterations in the properties of voltage-gated sodium channel currents in Jedi-1 null neurons. These results provide new insight into the expression pattern of Jedi-1 in the peripheral nervous system and indicate that loss of Jedi-1 alters DRG neuron activity indirectly through an intercellular interaction between non-neuronal cells and sensory neurons.

## Introduction

The primary afferent somatosensory neurons are a heterogeneous population of peripheral neurons responsible for detecting and transmitting information about external stimuli such as temperature, proprioception, touch, pain, and itch to the central nervous system (CNS), where they are integrated and processed^1^. These pseudo-unipolar neuron cell bodies reside in the dorsal root ganglia (DRG) and innervate peripheral tissues as well as extending axons into the CNS where they synapse in the dorsal horn of the spinal cord. Like the rest of the developing nervous system, approximately 50% of DRG neurons undergo normal developmental apoptosis^2^. The cellular corpses are efficiently removed through phagocytosis, which we previously demonstrated is carried out in the DRG by satellite glia, a specialized type of peripheral glial cell that enshrouds the sensory neuron somas^3^. This clearance process involved the phagocytic receptors MEGF10 and Jedi-1 (MEGF12/PEAR1), homologs of the well-characterized engulfment receptors Draper in *Drosophila melanogaster* and CED1 in *Caenorhabditis elegans*^3–5^.

Satellite glial cells (SGCs) envelop DRG neuron somas in very close proximity (~20 nm), an ideal anatomical position for their role in phagocytosis of apoptotic DRG neurons during embryogenesis. This close proximity also facilitates modulation of neuronal activity via several mechanisms, including the regulation of extracellular ion concentrations, the release of paracrine signaling molecules, and the creation of SGC nets linked through gap junctions^6^. SGCs, therefore, contribute to neuronal sensitization and chronic pain when they enter an ‘activated’ state, characterized by an increase in their proliferation, up-regulation of the intermediate filament protein glial fibrillary acidic protein (GFAP), and cytokine production^7^.

The DRG and the associated peripheral nerves contain a host of other cell types required for efficient conduction of electrical signals across long distances in the organism, including perineurial cells. These cells encapsulate the DRG and bundle groups of axons into fascicles within the nerve^8^. The canonical function of perineurial glia is to establish the blood-nerve-barrier (BNB) through tight junctions that occlude ions, molecules, and cells from entering the nerve^9^. Additionally, it has been reported that the perineurium can modulate the development of the neuromuscular junction (NMJ), Schwann cell myelin production, and aid in phagocytosis and regeneration after nerve injury^10–12^.

In the current study, we resolved to understand the role of Jedi-1 in the peripheral somatosensory nervous system by analyzing a global Jedi-1 knock-out (KO) mouse model. We first used wild type (WT) mice to characterize Jedi-1 expression in satellite glia and endothelial cells, where it has previously been detected^13,14^ and report that Jedi-1 is a novel marker for perineurial glia. We found no changes in morphology or function of peripheral glial cells in the absence of Jedi-1. Surprisingly, however, we did observe changes in DRG neuron activity, despite not detecting Jedi-1 protein in the neurons themselves. Specifically, in Jedi-1 KO neurons, there was an increase in the fraction of neurons responsive to capsaicin and patch-clamp electrophysiology revealed an increase in excitability, with a shift from phasic to tonic firing patterns in KO neurons. We also found alterations in the properties of voltage-gated sodium channel currents. These data indicate that Jedi-1 acts in a non-cell autonomous manner to modulate sensory neuron function.

## Methods

### Mice

All animal procedures were approved by the Vanderbilt University Medical Center’s comprehensive Animal Care and Use Program (ACUP) in compliance with the NIH guidelines for the Care and Use of Laboratory Animals. Mice were housed under a controlled 12-hour light/dark cycle. Standard laboratory rodent diet (*LabDiet* catalog no. 5001) and water was available *ad libitum*. Jedi-1 knock-out (KO) were Pear1^tm1a(KOMP)Wtsi^ mice derived from embryonic stem cells provided by the International Mouse Phenotype Consortium (IMPC, catalog no. CSD31459_C05). Control mice were wild-type (WT) C57/BL6 mice obtained from Jackson Labs and then maintained by our laboratory (catalog no. 000664).

### RT-PCR

RNA was isolated from flash frozen tissue using Trizol (ThermoFisher Scientific catalog no. 15596026) according to the manufacturer’s directions. The RNA was treated with TURBO DNase (Lift Technologies catalog no. AM2238). cDNA was made using an Invitrogen RT kit with oligo dT primers and RNase included (Invitrogen catalog No 18080-051). PCR amplification was performed using the primers specified below.

### Primers

The following primers were used to verify the orientation of the KOMP insert at the 5′ end of the Jedi-1 genomic DNA locus: All primer sequences listed 5′ to 3′: Forward, TCTGACCTCCTCTTGTGCCTC and reverse, GGCTTCACTGAGTCTCTGGCA. The following primers were used to verify the orientation of the KOMP insert at the 3′ end of the Jedi-1 genomic DNA locus: Forward, CTGCCACTGTCATAGCATTA and reverse, CACTTAATGACACTCCTTTC. The following primers were used to amplify mouse GAPDH transcript for RT-PCR: Forward, TGCACCACCAACTGCTTAG and reverse, GATGCAGGGATGATGTTC. The following primers were used to amplify mouse Jedi-1 transcript for RT-PCR: Forward, CCTGCAGCTGCCCACCGGGCTGGA and reverse, CCTGGCAGCCCGGGCCATGCGTGT.

### Immunoprecipitation (IP)/Westerns

#### Lysate preparation

Mouse tissue was flash frozen for western blots and stored at −80 **°**C. Tissue was ground into a fine powder then lysed and sonicated in RIPA buffer (1% NP-40, 0.5% deoxycholate, 0.1% SDS, 150 mM NaCl, 50 mM Tris-Cl pH 8.0) with protease and phosphatase inhibitors added according to the manufacturer’s specifications (Roche catalog no. 04693159001 and Roche catalog no. 04906837001).

#### For IP-westerns

Two mg total protein per samples was pre-cleared with 1:1 mixture of Protein A/G sepharose beads (Invitrogen catalog no. 101041 and 101241, respectively). Anti-Jedi rabbit polyclonal antibody^5^ (not commercially available) or control serum were incubated with the samples overnight at 4** °**C and pulled down with Protein A/G beads. After washing with lysis buffer, samples were denatured and run on an SDS-PAGE acrylamide gel, transferred to a nitrocellulose membrane, and blocked with 5% milk. IPs were immunoblotted with mouse anti-Jedi monoclonal^5^ (not commercially available) or mouse anti-Tubulin (Calbiochem catalog no. CP06) primaries and anti-mouse secondaries (Promega catalog no. W402B) and developed on an Amersham Imager 600 version 1.2.0. Some western blots were developed on film in a dark room.

#### For westerns

Similar to above but blotted with anti-Jedi1 (R&D catalog no. AF7607-SP) or anti-laminin (Millipore catalog no. AB2034).

### DRG cultures

DRGs from adult or early postnatal animals, as indicated in each experiment, were pooled from all levels and digested at 37 °C in 0.15% collagenase (Sigma catalog no. C5894), 0.05% trypsin (Worthington catalog no. LS003708), and 5 KU/mL DNase (Sigma catalog no. D5025) diluted in TESCA buffer (50 mM TES, 0.36 mM CaCl_2_, pH 7.4) for 5–30 minutes, depending on the efficiency of the digest. After inactivating the digest with Hyclone serum, cells were triturated with a fire polished glass pipette, spun at 100 × g for 6 minutes, and resuspended in media. Media used for DRG cultures: 1 to 1 mixture of Neurobasal (Gibco catalog no. 21103-049) and UltraCulture (Lonza catalog no. 12–725 F) media, 3% Hyclone serum (Hyclone catalog no. SH30088.03), 1% N2 (Gibco catalog no. 17502-048), 2% B27 (Gibco catalog no. 17504-044), 1% L-glutamine (Gibco catalog no. 25030-081), 1% Pen/Strep (Gibco catalog no. 15140-122), 50 ng/mL NGF (Harlan catalog no. B5017). Cells were plated on collagen (Sigma catalog no. C3867-1VL) coated glass coverslips and calcium imaging or patch clamping were completed within 24–48 hours after plating the cells.

### HeLa cell cultures

HeLa cells overexpressing Jedi-1 were used as a positive control for Jedi-1 staining *in vitro* and as a positive control for western blot. HeLa cells were maintained in DMEM (Gibco catalog no. 11995-065) with 10% serum (Peak catalog no. PS-FB2) and were transfected with a Jedi-1-GFP fusion construct made within our lab^5^ using Lipofectamine 2000 according to the manufacturer’s instructions (Thermo Fisher Scientific catalog no. 11668030).

### Immunohistochemistry (IHC)

#### General protocol

Tissue was fixed in 10% neutral buffered formalin (NBF) for 2 hours for small tissue and overnight for larger tissues. Samples were then dehydrated and embedded in paraffin. Five micron sections were cut. Tissues were then rehydrated and antigen retrieval performed using one of the following three methods: (1) Proteinase K (Macherey Nagel/Clontech Laboratores catalog no. 740506) at a final concentration of 20 micrograms/mL for 30 minutes at room temperature according to the manufacturer’s instructions. (2) Citrate buffer (10 mM citric acid, 0.05% Tween 20, pH 6.0) in pressure cooker for 12 minutes. (3) Tris-EDTA (10 mM Tris Base, 1 mM EDTA, 0.05% Tween 20, pH 9.0) in pressure cooker for 12 minutes. All washes were done with PBS. All tissue was blocked in 5% BSA, 0.1% Tween-20 diluted in PBS. Slides were mounted in ProLong Gold with DAPI (Life Technologies, catalog no. P36931). Regular fluorescence microscopy was performed on a Nikon Eclipse Ti microscope with a DS-Qi2 camera using NIS Elements AR version 4.5 software. Confocal images were acquired on a Leica SP5 confocal microscope using LAS AP software version 2.7.3.9723.

#### Statistical analysis

All microscopy images were analyzed using the open source processing software ImageJ version 2.0.0-rc-69/1.52p. Unless stated otherwise, we stained a minimum of 5 sections of ganglia or sciatic nerve at least 60 microns apart per animal for each measurement. Data points represent an average of repeated measurements per animal. Each animal used as a single ‘n’ for statistical analysis. The number of animals used for each experiment varies for each experiment and is reported in the figure legend or text. Statistical tests and graphs were performed and generated using Prism8 software version 8.3.

#### Primary antibodies used for IHC

PEAR1 (R&D catalog no. AF7607-SP), Laminin (Millipore catalog no. AB2034), Glut1 (Abcam catalog no. ab40084), BFABP (gift from Dr. Thomas Muller^15^), PGP9.5 (AbD Serotec catalog no. 7863-0504), GS (Santa Cruz catalog no. sc-6640-R), GFAP (Millipore catalog no. MAB360), HuC/D (Molecular Probes catalog no. A21272), ZO-1 (ThermoFisher Scientific, catalog no. 61-7300), Ki67 (Cell Signaling catalog no. 12202), TrpV1 (Alomone catalog no. ACC-030).

### Immunocytochemistry (ICC)

Cells were fixed in 10% NBF for 25 minutes at room temperature, permeabilized in 0.5% Triton-X-100 diluted in PBS for 5 minutes at room temperature, and blocked in 10% horse serum, 10% goat serum, 0.1% Tween-20 diluted in PBS for 1 hour at room temperature. Primary antibodies were diluted in blocking buffer and incubated on the cells overnight at 4 °C. Cells were washed with PBS and incubated with fluorescent secondary antibodies diluted in blocking buffer for 1 hour at room temperature. Cells were washed with PBS and mounted with 1 mm thick coverslips using ProLong Gold (Invitrogen catalog no. P36931) and imaged on a Leia SP5 confocal microscope at 100X magnification.

#### Primary antibodies used for ICC

anti-Jedi-1 (R&D catalog no. AF7607), anti-Tuj1 (Covance catalog no. 801213), anti-TrpV1 (Alomone catalog no. ACC-030). *Secondary antibodies used for ICC:* anti-sheep secondary (Abcam catalog no. ab175712), anti-mouse secondary (Thermo Fisher Scientific catalog no. A11029), anti-rabbit secondary (Invitrogen catalog no. A11035).

### Transmission electron microscopy (TEM)

Adult sciatic nerves were isolated and fixed in 0.1 M sodium cacodylate (Electron Microscopy Sciences [EMS], catalog no. 11652), 2% paraformaldehyde (EMS catalog no. 15713-S), 3% glutaraldehyde (EMS catalog no. 16310) for 1 hour at room temperature then overnight at 4 °C. Samples were washed three times in 0.1 M cacodylate buffer (wash buffer) and post-fixed in 1% osmium tetroxide (EMS catalog no. 19150) for 1 hour at room temperature then overnight at 0.5% osmium tetroxide. Samples were washed three times in wash buffer then gradually dehydrated with increasing concentrations of ethanol. Samples were then infiltrated in solutions with increasing ratios of Epon-812 (EMS catalog no. 14120) to propylene oxide (EMS catalog no. 20401) until reaching pure resin. Samples were embedded in Epon-812 and baked at 60 °C for 48 hours. Thick sections were cut at 0.5 microns on a Leica UC7 ultramicrotome and counterstained with toluidine blue. Thin sections were cut at 70–80 nm, counterstained with 2.0% uranyl acetate and Reynolds lead citrate, and collected on a 300 mesh copper grid. TEM images were acquired on a Philips/FEI T-12 microscope using an AMT CCD camera and AMT Image Capture Engine software version 600.156.

### Blood nerve barrier (BNB)

Sterile 2% Evan’s blue dye (Sigma catalog no. E2129) dissolved in PBS was injected intravenously. Three hours after the injection, mice were perfused with PBS and organs collected and placed in formamide. The samples were baked at 55 °C for 48 hours. Absorbance of the supernatant was measured at 610 nm and normalized to the weight of the tissue.

### Calcium imaging

Methods for calcium imaging were previously published and summarized here^16,17^. The fluorescent calcium (Ca^2+^) dye Fura-2 acetoxymethyl ester (AM) (Setarah Biotech catalog no. 6101) was used to measure cytosolic calcium concentration. Cells were washed twice with HEPES-buffered Hanks’ balanced salt solution (HBSS) and incubated for 30 to 45 min with 3 μM Fura-2AMat 37 °C and then washed in Fura-free HBSS solution for 30 to 60 min before recording. The coverslip with the cells attached was transferred to a recording chamber mounted on the stage of a Nikon TE2000 fluorescence microscope (Nikon Instruments Inc., Melville, NY). The total volume of the recording chamber was 300–400 uL and was constantly perfused from gravity-fed chambers at a rate of 4 mL/min with an NaCl-based extracellular solution containing 2 mM Ca^2+^ (145 mM NaCl, 2 mM KCl, 1 mM MgCl_2_, 10 mM glucose, 10 mM HEPES, 2 mM CaCl_2_, pH 7.3, ~305 mOsm). An InCyt IM2 fluorescence imaging system (Intracellular Imaging Inc., Cincinnati, OH) was used to monitor [Ca^2+^]_i_. Cells were alternately excited at wavelengths of 340 and 380 nm and emission at 510 nm detected using a PixelFly digital camera. Ratios were collected every 1 second throughout the experiment and converted to [Ca^2+^]_i_ using an i*n vitro* calibration curve, generated by adding 15.8 μM Fura-2 pentapotassium salt to solutions from a calibration kit containing 1 mM MgCl_2_ and known concentrations of Ca^2+^ (0–1350 nM) (Invitrogen). Cells with an unstable baseline were excluded from the analysis. Cells were treated with the various drugs for 30 or 60 seconds, followed by washout with the NaCl-based solution until calcium levels returned to baseline, except for capsaicin. Drugs used in the following order: 1 mM chloroquine (CQ) for 60 seconds, 100 uM histamine (HIST) for 60 seconds, 200 uM allylisothiocyanate (AITC) for 60 seconds, and 500 nM capsaicin (CAP) for 30 seconds. As a positive control, 90 mM KCl solution was added for 30 seconds to identify healthy neurons (57 mM NaCl, 90 mM KCl, 1 mM MgCl_2_, 10 mM glucose, 10 mM HEPES, 2 mM CaCl_2_, pH 7.3, ~305 mOsm). A cell was considered to respond to treatment if its intracellular calcium levels increased ≥15% above the baseline concentration in the 30 seconds prior to treatment. For cells that responded to capsaicin, their intracellular calcium concentrations remained high even after washout. Therefore, they often did not respond to KCl. As long as all of the other requirements were met, these cells were included in the analysis.

### Patch-clamp electrophysiology

All electrophysiology was performed on small diameter (<25 micron diameter) neurons maintained in culture for 24–48 hours following plating. All experiments were performed at room temperature (23–25 °C). Personnel performing patch clamp recording and initial data analysis were blinded to the genotype of the cells. Methods were similar to those we have previously described^16,18^. Patch pipette electrodes were pulled from borosilicate glass capillary tubes (World Precision Instruments, Sarasota, FL) using a Sutter P-97 pipette puller (Sutter Instrument, Novato, CA). Electrodes were coated close to the tip with dental wax (Electron Microscopy Sciences, Hatfield, PA) and fire-polished using a Narishige MF-830 microforge (Narishige, Amityville, NY) to a final resistance of ~2 MΩ when filled with patch-pipette solution. Cells were recorded in the conventional whole-cell configuration using a HEKA EPC10 amplifier and PatchMaster acquisition software (HEKA Instruments Inc., Holliston, MA, USA) or an Axopatch 200B amplifier, Digidata 1400 A interface, and PClamp10 (Clampex) acquisition software (Molecular Devices, Sunnyvale, CA). Series resistance was partially compensated (~50–80%) and analog data were filtered at 10 kHz and digitized at 100 kHz for voltage-clamp recording of fast voltage-gated sodium channel currents, or filtered at 5 kHz and digitized at 20 kHz for current clamp recording.

For current clamp recording, individual cells were first voltage-clamped and allowed to stabilize (patch solution equilibrate) for approximately 3–4 minutes. Membrane capacitance was determined using Patchmaster software as an objective measure of cell size (surface area). The recording mode was then switched to current clamp and spontaneous membrane potential was recorded for 1–2 minutes. Cells with an unstable resting membrane potential, or resting potential more depolarized than −45mV were discarded. Membrane resistance was determined by applying small (5–10 pA, 100 ms) hyperpolarizing and/or depolarizing current steps and recording the resulting change in membrane potential. Cells were stimulated with a series of 100 ms current steps of increasing magnitude (increment 5–10pA) to determine rheobase (the smallest current step that evoked an action potential). The cell was then stimulated with a 1 second step depolarization first to rheobase and then to twice rheobase to determine the number and pattern of evoked action potentials. Phasic firing was defined as a cell that fired one or two action potentials within the first 100 ms of stimulation and then remained silent for the remainder of the stimulus. Tonic firing was defined as 3 or more action potentials occurring throughout the duration of the stimulus. Action potential parameters were determined from the first evoked action potential and are summarized in Table 1. The threshold potential was determined by calculating the 1^st^ derivative of the action potential to better identify the onset of the upstroke of the action potential. Threshold potential was empirically set to be the potential at the time point when dV/dt reached 10% of its maximum value.

Voltage-clamp data for *I*_Na_ were subjected to linear capacitance and leak subtraction using P/N protocols (P/−4 or P/−8) with the leak pulses applied following the test pulses. The recording bath (volume ~300 uL) was continually perfused with fresh solution at a flow rate of ~3–4 ml/min from gravity-fed reservoirs. Standard extracellular recording solution (see below) was the same as that used for current clamp. In some cells extracellular NaCl was replaced with TEA-Cl to confirm the fast voltage-gated current was carried primarily by sodium channels. The voltage protocols are shown on the figures. Conductance was calculated from the peak inward current evoked by a voltage step as follows: G = I/(V – E_Na_), where G = conductance, I = peak sodium current, V = membrane potential of the voltage step and E_Na_ = the reversal potential of sodium channel current).

Solutions used for electrophysiology were as follows: extracellular recording solution contained 145 mM NaCl, 2 mM KCl, 1 mM MgCl_2_, 10 mM glucose, 10 mM HEPES, 2 mM CaCl_2_, pH 7.3, ~315–320 mOsm adjusted with sucrose as needed. The intracellular patch pipette solution for current clamp contained 140 mM KCl, 0.5 mM EGTA, 5 mM HEPES, 3 mM Mg-ATP, pH 7.3, ~305–310 mOsm adjusted with sucrose as necessary. The intracellular patch pipette solution used for voltage-clamp recording of I_Na_ contained 140 mM CsCl, 10 mM NaCl, 2 mM EGTA, 10 mM HEPES, 2 mM MgCl_2_, 2 mM Mg-ATP, pH 7.3, ~305–310 mOsm adjusted with sucrose as necessary. The calculated liquid junction potentials were ~5 mV and were not corrected. All experiments were performed at room temperature (~23–25 °C). Raw data were analyzed using PClamp10 (Clampfit) or HEKA FitMaster software. Graphing and statistical analyses were performed using OriginPro 2016.

## Results

### Validation of mouse model

In order to study the role of Jedi-1 *in vivo*, we used a knock-out (KO) mouse model available through the Knockout Mouse Model Project (KOMP) repository (https://www.komp.org) (Supplementary Fig. S1A). Through PCR of genomic DNA, we verified the correct location and orientation of the KOMP construct (data not shown) and by RT-PCR, validated that full-length Jedi-1 mRNA was not expressed in Jedi-1 KO mice (Supplementary Fig. S1B). Using two antibodies against the intracellular domain of Jedi-1, we also confirmed that Jedi-1 KO mice do not express Jedi-1 protein (Supplementary Fig. S1C). Additionally, we did not detect the N-terminal fragment of Jedi-1 using a polyclonal antibody generated against the entire extracellular domain (Supplementary Fig. S1D).

### Jedi-1 is expressed in peripheral nervous system glia

**Figure 1:**
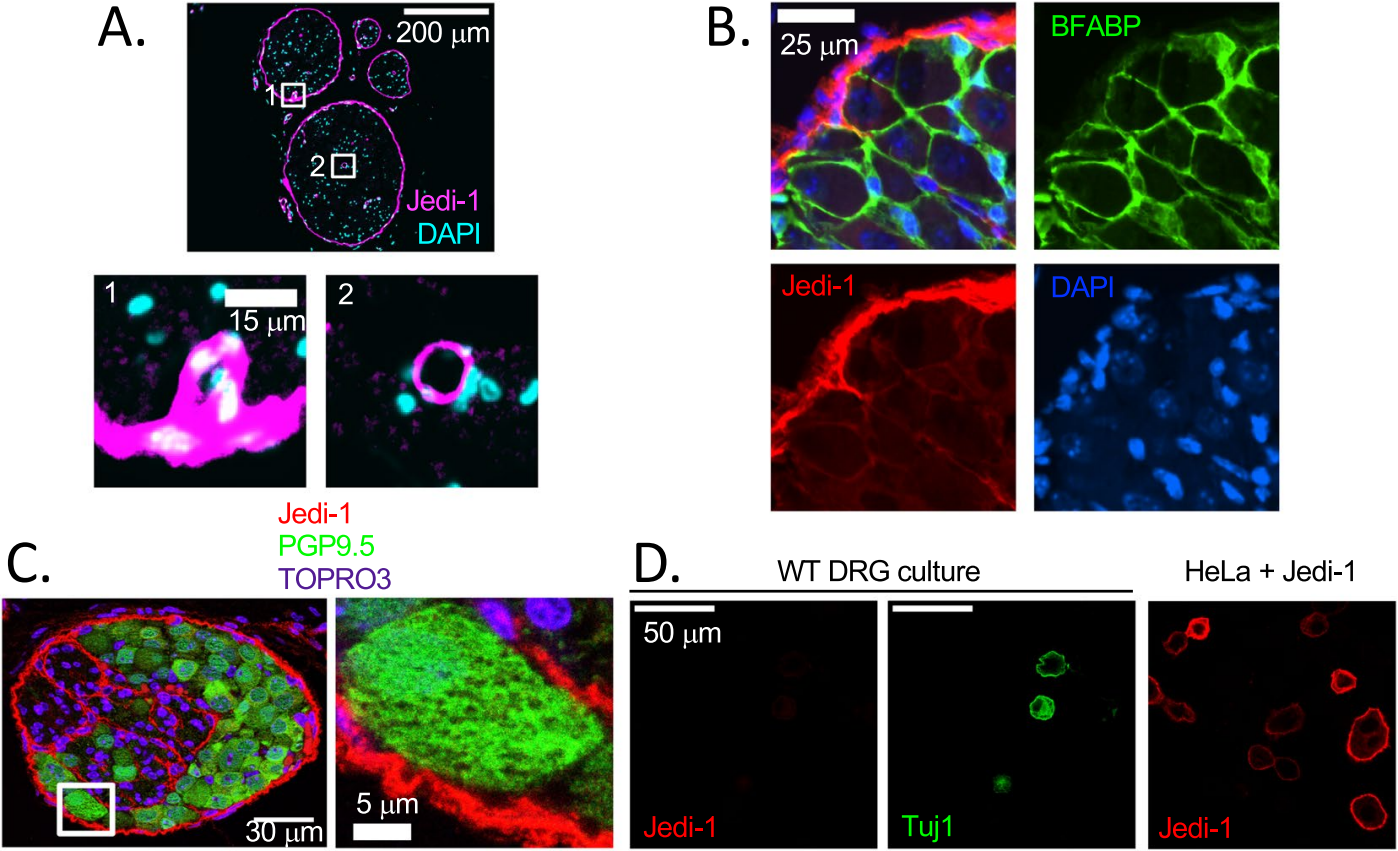
Jedi is expressed in peripheral glia and endothelium. (**A**) Cross section of adult (8–12 weeks old) WT sciatic nerve stained for Jedi-1 (magenta) and DAPI (blue). Insets 1 and 2 show Jedi expression in blood vessels. See Supplementary Fig. S2 for validation of Jedi-1 expression in perineurial glial cells. (**B**) WT P0 DRG co-stained for Jedi-1 (red), satellite glial marker BFABP (green), and DAPI (blue). (**C**) WT DRG co-stained for Jedi-1 (red) and PGP9.5 (green) and TOPRO3 (blue). Right shows inset. (**D**) Right: Primary WT DRG cultures stained for neurons using Tuj1 (green) and Jedi-1 (red). Left: HeLa cells overexpressing mouse Jedi-1 were used as a positive control for Jedi-1 immunocytochemistry *in vitro*. All images were analyzed in ImageJ version 2.0.0-rc-69/1.52p.

We previously reported that Jedi-1 mRNA is expressed in the dorsal root ganglia (DRG) both *in vivo* and in primary DRG mixed neuron-glia co-cultures^3^. According to the Broad Institute resource Genotype-Tissue Expression (GTEx) human RNAseq database, the highest level of Jedi-1 expression is in peripheral nerves (https://gtexportal.org/home/), so we investigated Jedi-1 protein expression in the context of the peripheral nervous system with a focus on the sciatic nerve. Consistent with previous reports^13,14^, we confirmed Jedi-1 expression in structures that appear to be blood vessels (Fig. 1A insets). In addition, we detected Jedi-1 expression in the perineurium of the nerve (Fig. 1A and Supplementary Fig. S2). We confirmed Jedi-1 expression in the perineurium by staining adjacent sections for Glut1, a marker for the perineurium as well as blood vessels^19^ (co-staining was not possible due to antibody incompatibility) (Supplementary Fig. S2A). We further verified Jedi-1 expression in perineurial cells using several other markers, including laminin, Claudin and ZO-1^20^ in both the nerve and ganglia (Supplementary Fig. S2B and S2C). To our knowledge, this is the first report showing that Jedi-1 is a perineurial marker.

In addition to nerve samples, we also stained DRGs for Jedi-1 and again found the highest levels of expression in the perineurium (Fig. 1B). Jedi-1 was also expressed at lower levels in a subset of satellite glial cells (SGCs), as detected by BFABP staining (Fig. 1B). We also checked whether Jedi-1 was expressed in DRG neurons by co-staining for Jedi-1 and PGP9.5, a pan-neuronal marker, *in vivo* (Fig. 1C) and *in vitro* by co-staining WT DRG cultures with Tuj1 (Fig. 1D). However, we did not observe any Jedi-1 signal in the neurons. In total, these results indicate that Jedi-1 is expressed in peripheral glial and endothelial cells but not in DRG neurons.

### Peripheral glial cell morphology and function are not altered in the absence of Jedi-1

**Figure 2:**
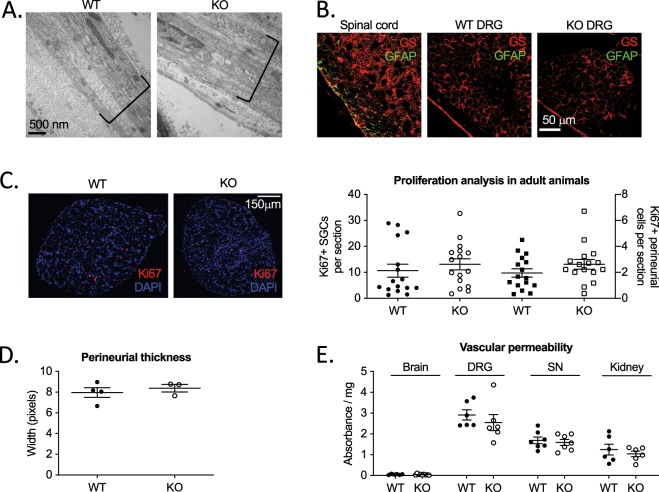
Peripheral glia are not altered in the absence of Jedi-1. (**A**) TEM images of WT and KO sciatic nerves showing the morphology of the perineurial cell layer, labeled in brackets. (**B**) Glutamine synthetase (GS, red) and glial fibrillary acidic protein (GFAP, green) immunostaining of spinal cord (positive control) or DRG of P1 mice from WT and KO animals. (**C**) Ki67 (red) and DAPI (blue) staining in adult WT or KO DRG 8–12 weeks old. Circles (left axis) are Ki67+ SGCs while squares (right axis) are Ki67+ perineurial cells. 16 animals analyzed per genotype with a minimum of 5 sections 60 microns apart analyzed per animal. No statistical difference between genotypes. (**D**) Quantification of laminin IF pictures such as those shown in Supplementary Fig. S2C. Thickness of the perineurial sheath measured in pixels. n = 3 animals analyzed per genotype with a minimum of 8 sections analyzed per animal at least 60 microns apart. Error bars represent SEM. No statistically significant difference between genotypes. (**E**) Evan’s blue dye extravasation from brain, DRG, sciatic nerve (SN), and kidney in adult WT and KO mice. Absorbance normalized to weight of tissue. No statistical difference between genotypes. All images were analyzed in ImageJ version 2.0.0-rc-69/1.52p.

Since we observed expression of Jedi-1 in the perineurium and the SGCs, we investigated whether the KO animals exhibit changes in the development of these peripheral glia cell types. H&E staining of paraffin sections (Supplementary Fig. S3A) and transmission electron microscopy (TEM) of the sciatic nerve (Fig. 2A) show normal morphology of the perineurial cell layer. In Jedi-1 KO animals there was no sign of glial activation in the SGCs based on glutamine synthetase (GS) and glial fibrillary acidic protein (GFAP) staining in early post-natal animals (Fig. 2B) and one-year-old animals (data not shown).

Previous reports indicate that Jedi-1 is a negative regulator of proliferation in endothelial cells lines^14^, bone marrow mononuclear cells^21^, and megakaryocyte precursors^22^. However, we did not find any significant difference between genotypes in the proliferation rate of SGCs or perineurial cells based on Ki67 staining (Fig. 2C). Because a change in perineurial proliferation might lead to thickening of the perineurial barrier around the nerve, we measured width of the perineurium (Fig. 2D), but found no change in Jed-1 KO mice. The perineurium also produces extracellular matrix (ECM), which we measured by western blot for collagen IV (data not shown) and laminin (Supplementary Fig. S3B), but found no significant change in ECM levels at several developmental ages.

The perineurium and peripheral nerve endothelial cells both contribute to the blood nerve barrier, which shields the endoneurial space from extreme changes in solute concentration and protects axons from potentially cytotoxic substances^23^. Since both of these cell types express Jedi-1, we tested the integrity of the blood nerve barrier by measuring extravasation of Evan’s Blue dye from the blood to the nerve and sensory ganglia, but we did not detect a significant difference in permeability between genotypes (Fig. 2E). As a control for a tissue with limited permeability, we measured Evan’s Blue in the brain, and as a positive control for tissue penetration of the dye, we measured kidney. In both cases, we found no change in Jedi-1 KO mice relative to WT control animals (Fig. 2E).

Previous studies from our lab demonstrated that SGCs phagocytose apoptotic neurons during the development of the DRG and *in vitro* evidence indicated that this occurs via Jedi-1-dependent engulfment^4,5^. However, when we performed TUNEL staining at E13.5, during the peak of apoptosis, and P1, after the normal cell death period^24^, we did not observe an accumulation of dead cells in Jedi-1 KO DRGs relative to WT (Supplementary Fig. S3C). We propose that other phagocytic receptors may compensate for lack of Jedi-1 such as MEGF10, Tyro3, Axl, or Mer. In summary, we did not detect any morphological or functional differences in the perineurium or SGCs in the absence of Jedi-1 under basal conditions.

### Altered functionality of sensory neurons from Jedi-1 KO mice

Although we did not detect Jedi-1 in DRG neurons, we considered the possibility that Jedi-1 deficiency in glial cells could indirectly affect the neurons. The *Drosophila* homolog of Jedi-1, Draper, is expressed by glia - not neurons - yet deficiency in Draper leads to neurodegeneration^25^. In addition, SGCs and endothelial cells, where we detected Jedi-1 expression, can release factors that affect sensory neuron function^6^. We also detected high levels of Jedi-1 in perineurial cells, which have been shown to be important for axon regeneration in zebrafish and mammals^10,12^. Therefore, we investigated DRG neuron function in Jedi-1 KO mice.

**Figure 3:**
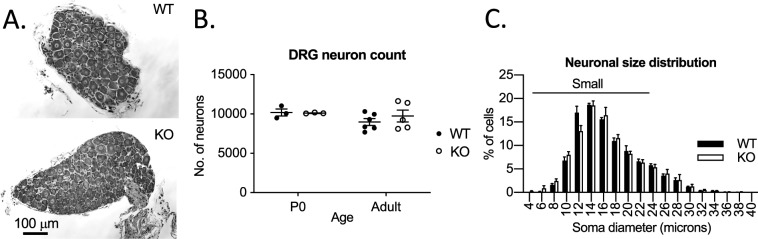
DRG neurons develop normally in the absence of Jedi-1. (**A**) Example pictures of toluidine blue staining of DRG sections from adult (8–12 weeks) WT and KO mice quantified in B and C. (**B**) Quantification of total DRG neuron counts from serially sectioned thoracic level 13 (T13) DRGs where every 12th section was counted, summed, and total neuron counts were estimated by multiplying by 12. Analysis was performed at two different developmental ages, P0 and adult animals 8–12 weeks old. Error bars indicated SEM. No statistically significant change between genotypes at either time point. (**C**) Frequency distribution of soma size measured from toluidine blue staining of adult DRGs WT n = 4 animals, KO n = 3 animals with a minimum of 500 cells analyzed per animal. Small DRGs less than 25 microns in diameter are indicated by the top horizontal bar. Error bars indicated SEM. No statistical difference between genotypes in any group using 2-way ANOVA. All images were analyzed in ImageJ version 2.0.0-rc-69/1.52p.

We did not find any loss of sensory neurons in the Jedi-1 KO mice (Fig. 3A,B) or alteration in size distribution, which correlates with sensory modality (Fig. 3C). The number of neurons per ganglion varies with spinal level. Thus, to enable direct comparison between genotypes, we quantified cell number and size in thoracic level 13 (T13) ganglia as this spinal level is readily identifiable. We suggest our data from T13 is representative of all ganglia; however, it is possible that other DRGs may have differences not represented in T13.

**Figure 4:**
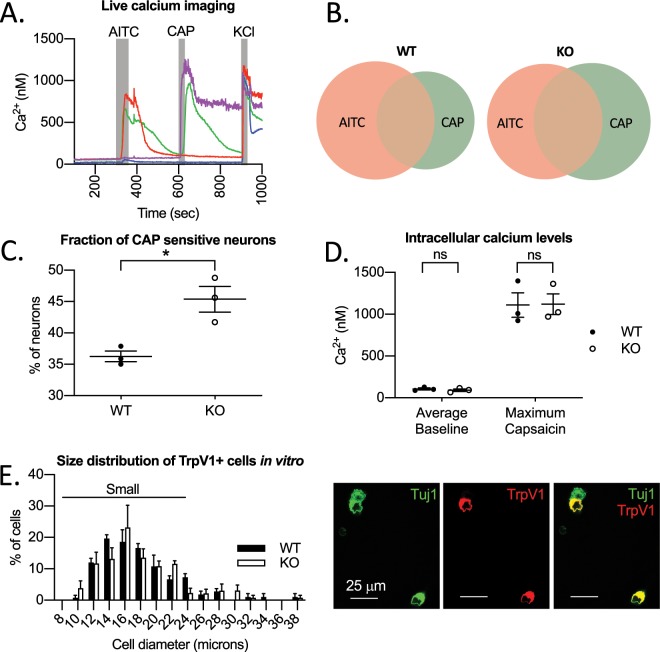
DRG neurons isolated from Jedi-1 KO mice are sensitized to capsaicin. (**A**) Representative trace of 4 cells used to obtain live cell calcium imaging data. Duration of treatment with allylisothiocyanate (AITC), capsaicin (CAP), and KCl is shown in grey boxes. Blue cell is a healthy neuron that responded to KCl but not AITC or CAP. Red cell responded to AITC and KCl. Purple cell responded to CAP and KCl. CAP responding cells often did not return to baseline. Green cell responded to AITC, CAP, and KCl. (**B**) Venn diagrams showing the proportions of cells that responded to AITC and CAP. t tests were used to compare overall responses to each drug. There was no statistical difference in AITC responses between genotypes. (**C**) Fraction of cells that responded to capsaicin over the course of 3 independent experiments with a minimum of 100 cells analyzed in each experiment. Two-tailed t test with Welch’s correction p = 0.03. (**D**) Intracellular calcium concentration measured by Fura-2AM. 34. Baseline calcium concentration was averaged over the course of 1 minute before any drug treatment. Maximum calcium concentration is the peak calcium concentration during treatment with 500 nM capsaicin for 30 seconds. Each data point represents an independent experiment with a minimum of 100 cells analyzed in each. Students t test was used to compare calcium concentrations between genotypes at baseline and after CAP treatment. (**E**) Left: Quantification of the size of of TrpV1+ neurons *in vitro*. n = 4 animals per genotype analyzed. Scale bars represent SEM. 2 way ANOVA used to compare genotypes across all size categories. No statistical differences between genotypes. Right: Representative image of TrpV1 staining in primary DRG cultures showing neurons in Tuj1 (green) and TrpV1 (red). All images were analyzed in ImageJ version 2.0.0-rc-69/1.52p.

To assess neuronal function, we performed live cell imaging of cultured DRG neuron calcium responses to several chemical pruritogens and allogens including chloroquine, histamine, allyl isothiocyanate (AITC), or capsaicin (CAP) (Fig. 4A). Although this group of compounds is not comprehensive for all DRG neuron subtypes, the vast majority of DRG neurons (70% by our measure) will respond to one or more of these drugs. We found that the percentage of cells that responded to chloroquine, histamine, and AITC were not statistically significant between genotypes. However, the proportion of cells that responded to capsaicin, a TrpV1 ion channel agonist, was increased in cells isolated from Jedi-1 KO mice (Fig. 4B,C). The baseline calcium concentration was not statistically different between genotypes, nor was the maximum intracellular calcium concentration following capsaicin treatment (Fig. 4D).

These results indicate that loss of Jedi-1 indirectly affects the fraction of DRG neurons responding to capsaicin, which suggests that either there is an alteration in the distribution of neuron subtypes or that TrpV1 is up-regulated or its activity sensitized. It is interesting to note that such up regulation and/or sensitization of TrpV1 occurs in inflammatory pain^26^. To investigate TrpV1 expression, we used immunostaining of both intact fixed ganglia and cultured DRG neurons (Fig. 4E). We did not find a significant change in the fraction of cells that express TrpV1 in the intact ganglia (WT 29.6% ± 4.9 SD of HuC/D+ neurons stained positive for TrpV1 compared to KO 28.3% ± 2.0 SD, n = 3 animals per genotype) or in isolated cells (WT was 32.5% ± 4.3 SD of Tuj1+ neurons compared to KO 29.2% ± 3, n = 4 animals analyzed per genotype; no significant changes between genotypes using Student’s t test for both data). The size distribution of TrpV1+ cells was also not significantly different between genotypes (Fig. 4E). Note that the majority (90%) of TrpV1+ cells were small diameter, which we defined as <25 microns (Fig. 4E). The analysis of TrpV1 expression suggests that Jedi-1 KO neurons have not changed their subtype distribution or identity; rather, TrpV1 sensitivity is being modulated through a mechanism that does not involve an increase in total TrpV1 protein levels. There are a number of post-translational modifications that could account for this, such as phosphorylation or glycosylation^27^.

### Increased excitability of Jedi-1 KO neurons

**Figure 5:**
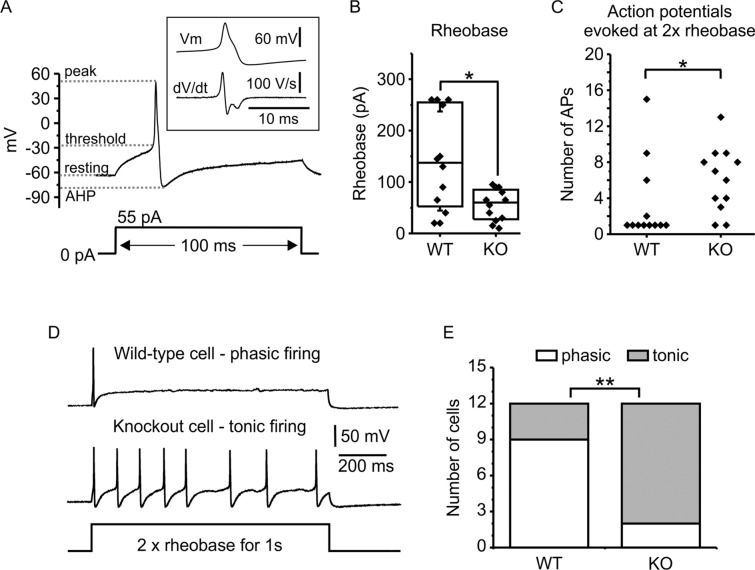
DRG neurons cultured from Jedi-1 KO mice are hyperexcitable. Patch clamp electrophysiology was used to record evoked action potentials from small diameter WT and KO DRG neurons. (**A**) Representative current-clamp recording from a Jedi-1 KO neuron. Stimulation with an excitatory current step (55 pA for 100 ms, lower trace) evoked a typical action potential (upper trace). The upper inset trace shows the action potential on an expanded time scale along with the 1st derivative of the trace. (**B**) Rheobase (defined as the smallest current step that evoked an action potential) was significantly smaller in Jedi-1 KO cells (*p = 0.03, Mann-Whitney test). Each point is from an individual cell and the box indicates median, 25% and 75% of the distribution. (**C**) Each point represents the number of action potentials evoked in an individual cell by a 1s current step at twice rheobase (see panel D). Significantly more action potentials were evoked in Jedi-1 KO neurons compared to WT (*p = 0.04, Mann-Whitney test). (**D**) Representative traces from a WT neuron (upper trace) and Jedi-1 KO neuron (lower trace) stimulated with a 1s current step at twice rheobase. The WT cell displayed phasic firing (a single action potential evoked at the onset of the stimulus) and the KO cell displayed tonic firing (8 action potentials evoked over the duration of the 1s stimulus). (**E**) The number of cells that displayed either phasic or tonic firing during a 1s stimulus (as in panel D) is shown. The proportion of Jedi-1 KO cells displaying tonic firing was significantly higher than WT (**p = 0.01, Fishers exact test).

As another approach to assess alterations in neuronal activity, we used whole cell patch-clamp electrophysiology. We first compared the basic membrane properties and excitability of small diameter neurons (<25 microns, which are primarily nociceptors) isolated from WT and KO mice using current-clamp recording (Fig. 5A). The membrane capacitance, which is directly proportional to the surface area of the cell, was not significantly different between genotypes, confirming neurons were of similar size (WT 19.9 ± 11.1 (SD) pF, n = 12; KO 18.4 ± 11 (SD) pF, n = 12, p = 0.75). Membrane resistance was slightly higher and resting membrane potential was slightly more depolarized in KO cells, but neither of these parameters were significantly different (Table 1). To test membrane excitability we applied a series of 100 ms depolarizing current steps of increasing magnitude. Rheobase, defined as the smallest current step that evoked an action potential, was significantly less in KO neurons, indicating increased excitability compared to WT (p = 0.03) (Fig. 5A, B). We compared various parameters of the evoked action potentials in KO and WT neurons (Table 1). The threshold potential was slightly more depolarized (p = 0.048), the after hyperpolarization was larger (p = 0.009), and the overall amplitude of the action potential was larger in KO compared to WT neurons (p = 0.027). There was no significant difference in the peak membrane voltage (p = 0.065), the rising slope (p = 0.32), or the duration at half maximal amplitude (half width) (p = 0.48) of the action potential (Table 1). The most overt difference between genotypes was the number and pattern of action potentials evoked when neurons were stimulated using a current step to twice rheobase for 1 second (Fig. 5C,D). The KO neurons fired significantly more action potentials compared to WT neurons (Fig. 5C, p = 0.04). Moreover, there was a significant (p = 0.01) shift in the firing pattern of KO neurons; 10 out 12 KO neurons displayed a tonic firing pattern with action potentials evoked throughout the duration of the stimulus. In contrast, only 3 out 12 WT neurons displayed tonic firing and the remaining 9 cells displayed phasic firing in which one or two action potentials were evoked at the start of the stimulus and the cell then remained quiescent (Fig. 5D,E).

**Table 1:** Basic electrical properties and parameters of evoked action potentials from WT and Jedi-1 KO neurons. Properties of WT neurons (n = 12 cells from 4 independent preparations) or Jedi-1 KO neurons (n = 12 cells from 3 independent preparations) are shown along with results of statistical comparisons performed using Student’s t-test or Mann-Whitney test for parametric and non-parametric datasets, respectively. Bold font indicates properties that were significantly different in Jedi cells compared to wild-type (* p < 0.05, ** p < 0.01).

	WT (n = 12)	KO (n = 12)	Statistical comparison
Membrane capacitance (pF) [mean (SD)]	19.9 (SD 11.1)	18.4 (SD 11)	p = 0.75 (t test)
Membrane resistance (MΩ) [median (1^st^, 3^rd^ quartile)]	387 (168, 612)	576 (366, 820)	p = 0.24 (Mann-Whitney test)
Resting membrane potential (mV) [mean (SD)]	−61.5 (SD 6.9)	−57.2 (SD 4.2)	p = 0.08 (t test)
**Rheobase (pA)** [median (1^st^, 3^rd^ quartile)]	138 (46, 258)*	**60 (26, 88)**	**p** = **0.03 (Mann-Whitney test)**
AP threshold potential (mV) [mean (SD)]	**−24.5 (SD 5.8)**	−20 (SD 4.7)*	**p** = **0.048 (t test)**
Membrane potential at peak (mV) [mean (SD)]	51 (SD 10)	57 (SD 5)	p = 0.065 (t test)
Maximal rising slope (V/s) [mean (SD)]	162 (SD 76)	138 (SD 30)	p = 0.32 (t test)
Membrane potential at peak of afterhyperpolarization (mV) [mean (SD)]	−73 (SD 5.4)	−77 (SD 3.5)	p = 0.056 (t test)
**Amplitude of afterhyperpolarization (change from baseline) [mean (SD)]**	**11.8 (SD 8.3)**	19.9 (SD 4.3)**	**p** = **0.009 (t test)**
**Action potential amplitude (peak to AHP) [mean (SD)]**	**124 (SD 12.6)**	134 (SD 7)*	**p** = **0.027 (t test)**
AP duration at half max (ms) [mean (SD)]	2.0 (SD 0.46)	1.9 (SD 0.3)	p = 0.48 (t test)

**Figure 6:**
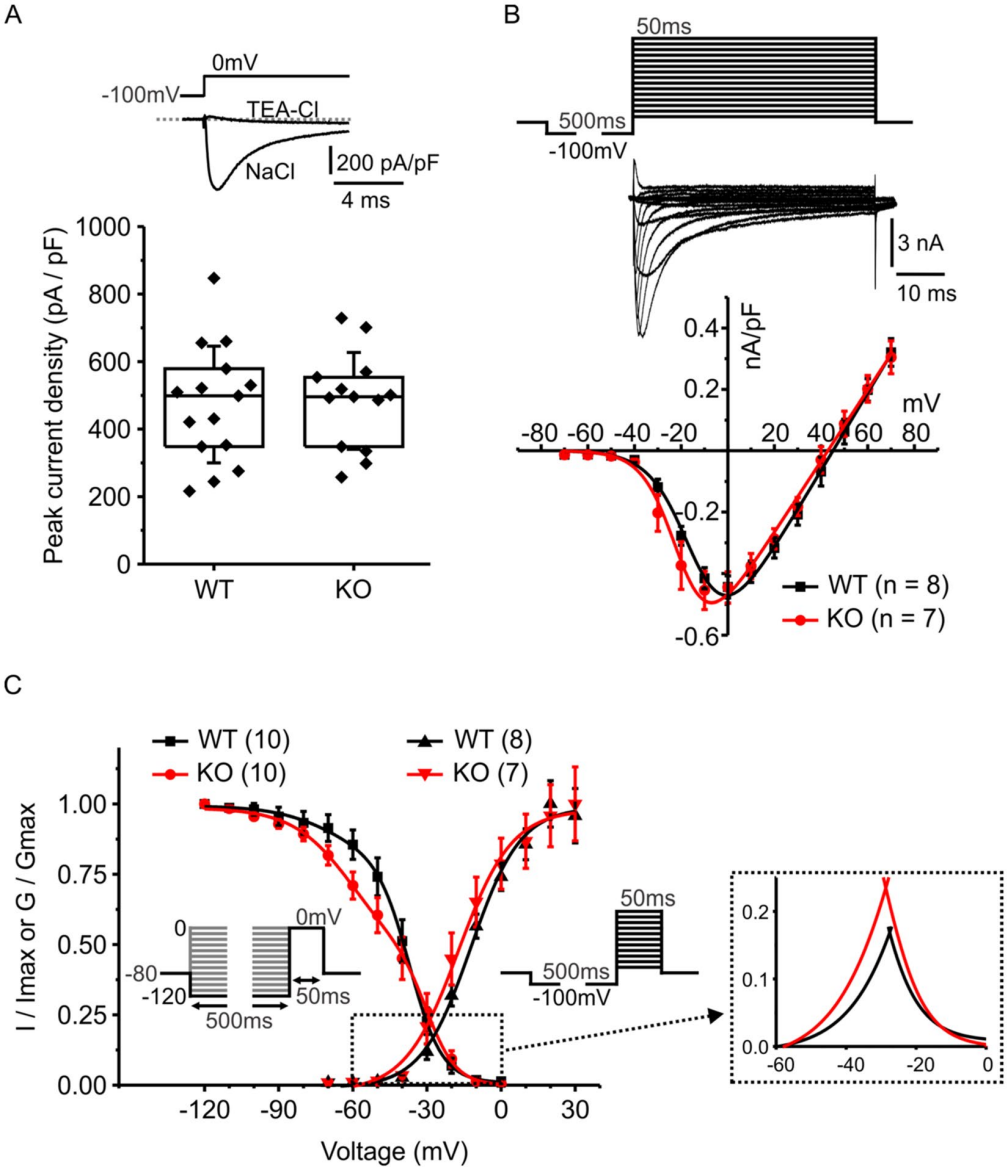
Voltage gated sodium channels have altered properties in DRG neurons isolated from Jedi-1 KO mice. Whole cell patch clamp electrophysiology was used to record voltage-gated sodium channel currents from small diameter wild-type (WT) and Jedi-1 knockout (KO) DRG neurons. (**A**) The upper trace shows two superimposed currents from the same cell evoked by a voltage step from −100 mV to 0 mV. Replacement of extracellular NaCl with TEA-Cl abolished the fast inward current confirming it was due to activation of voltage-gated sodium channels. The peak current density evoked by a voltage step to 0 mV was not significantly different between genotypes. Each point is from an individual cell, box indicates median, 25% and 75% of the distribution, whisker indicate standard deviation of the mean. (**B**) Current-voltage relationship for wild type (WT) and knockout (KO) neurons. The upper panel shows an example of the stimulus protocol and sodium currents from a representative neuron. The lower panel plots mean peak current density against the test potential. (**C**) Normalized inactivation curves (left two curves) and activation curves (right two curves) for wild type and knockout neurons are superimposed. The inset cartoons depict the voltage protocols: inactivation was produced by a series of 500ms steps (−120 mV to 0 mV) prior to a 50ms test pulse to 0 mV. Peak current amplitude produced by the test pulse was normalized to the largest current and plotted against the voltage of the 500ms conditioning pulse. Solid curves show the fit with a double Boltzmann function. Activation curves were derived from the same data used to produce the current voltage-relationship in panel (B) (see methods for more detail). Solid curves show the fit with a Boltzmann function. The “window current” is shown on an expanded scale in the dashed box to the right.

These results indicate that Jedi-1 KO neurons are hyper-excitable, suggesting there might be differences in the expression and/or function of ion channels. To investigate this further we used voltage clamp recording, focusing on voltage-gated sodium channel currents (*I*_*Na*_). The peak sodium current density (evoked by a voltage step to 0 mV) was not significantly different in KO neurons compared to WT neurons (Fig. 6A). The current-voltage relationships (Fig. 6B) were similar for the two genotypes, with a slight (4–5 mV) hyperpolarizing shift apparent in the KO neurons that was also apparent in the activation curves (plotting normalized conductance Vs voltage) (Fig. 6C). However, the midpoint of activation (V_50_) determined by fitting each individual cell was not significantly different (WT −12.8 ± 4.4 (SD) mV, n = 8; KO −17.5 ± 7.4 (SD) n = 7, p = 0.14). Steady state inactivation (Fig. 6C) was best fit using a double Boltzman function and there was a significantly larger contribution of the more hyperpolarized component in KO compared to WT neurons (Table 2). The inactivation and activation curves are plotted together in Fig. 6C showing the shifts in voltage-dependence of *I*_*Na*_ result in a slightly larger and hyperpolarized window current in KO neurons. Overall, these data support the idea that changes in the expression and/or function of voltage-gated sodium channels contribute to the increased excitability of KO neurons.

**Table 2:** Activation and inactivation parameters of I_Na_. Parameters of the Boltzman fits to the activation and inactivation curves. Each individual cell was fit with a single (activation) or double (inactivation) Boltzman function and the indicated parameters pooled for statistical comparison using Student’s independent t-test. V_50_ act is the midpoint at which 50% of the channels were activated. V_50_ inact are the midpoints for the two components of the inactivation curve. *denotes statistically significant difference, p < 0.05.

Activation	WT (n = 8)	KO (n = 7)	
V_50_ act (mV)	−12.8 (SD 4.4)	−17.5 (SD 7.4)	p = 0.15
Slope (activation) (mV)	9.6 (SD 1.4)	8.5 (SD 2.4)	p = 0.29
**Inactivation**	**WT (n = 10)**	**KO (n = 10)**	
V_50_ inact 1 (mV)	−68 (SD 13.5)	−70 (SD 10.7)	p = 0.77
V_50_ inact 2 (mV)	−38 (SD 7.8)	−33 (SD 10.1)	p = 0.24
Slope inact 1 (mV)	13.5 (SD 6.3)	15 (SD 7.8)	p = 0.69
Slope inact 2 (mV)	5.4 (SD 1.1)	5.6 (SD 1.6)	p = 0.71
Fractional contribution of component 1	0.17 (SD 0.17)	0.39 (SD 0.27)	*p = 0.046

## Discussion

DRG neuron hyperexcitability is correlated with chronic pain, a severe problem in our society that affects an estimated 50 million people in the United States alone^28^. Understanding the cellular and molecular mechanisms that regulate neuro-responsiveness is essential for improved treatment of chronic pain conditions. In the current study, we demonstrate that loss of the phagocytic receptor Jedi-1 increases DRG neuronal excitability; however, Jedi-1 was not detected in the neurons, but was expressed by endothelial cells, satellite glia and perineurial cells. These results indicate that deficiency in Jedi-1 indirectly alters sensory neuron activity in a non-cell autonomous mechanism. Our findings contribute to the growing body of literature that suggests that neuron-glia interactions are essential for proper regulation of neuronal homeostasis.

We found that satellite glial cells (SGCs) express low levels of Jedi-1, similar to previous results^3^. Satellite glial cells have a well-established role in augmenting DRG neuron activity through several mechanisms, including paracrine signaling and regulation of extracellular ion concentration^6^. SGCs release a number of soluble factors that contribute to the inflammatory milieu including PGE2^29^, IL1-β^30^, and TNFα^31^. Conversely, the SGCs respond to signals from the neurons including neurotransmitters and neuropeptides, which act in a feed forward mechanism to produce more inflammatory signals from the SGCs. SGCs typically exhibit canonical signs of activation when they enhance neuronal activity and potentiate chronic pain, which includes increased proliferation and up-regulation of GFAP. In the current study, we did not observe morphological changes or traditional activation markers to satellite glia. It is possible that Jedi KO SGCs operate through a novel mechanism independent of conventional activation pathways or that Jedi-1 acts during a specific developmental window that was not observed in our analysis focused mostly on postnatal mice.

Surprisingly, loss of Jedi-1 did not result in the accumulation of apoptotic neurons, despite our previous *in vitro* findings that Jedi-1 contributed to the phagocytosis of neuronal corpses by satellite glia^3^. We suggest that this is due to the presence of additional engulfment receptors; for example, MEGF10 is also expressed by these glia^3^. Recent findings suggest that satellite glia are developmentally arrested Schwann cells^32^ and Schwann cells have been shown to express multiple engulfment receptors^33^. Therefore, it is likely that a phagocytic deficiency will only be detected by deletion of multiple engulfment receptors.

Jedi-1 was also detected in endothelial cells, in agreement with previous studies^13,14,34^. The endothelium lies in very close proximity to the neurons and can influence their function directly by producing pro-nociceptive compounds or indirectly by attracting inflammatory cells such as macrophages and T cells^35–37^. In rodent models of chronic pain including nerve injury and chronic inflammation, the vasculature undergoes neoangiogenesis, increases in branching complexity, and has a distinct ‘leakiness’ believed to contribute to chronic pain^38,39^. In our Jedi-1 KO animals, however, we did not find changes in vascularization in the ganglia or nerve (data not shown), nor did we observe differences in leakiness of the vessels (Fig. 2E). Our results confirm a prior study that utilized the same Jedi-1 KO mouse model to show no significant changes in baseline density of blood vessels in muscle and skin^14^. Therefore, while the Jedi-1 deficient endothelial cells could be responsible for the altered neuronal activity, it is not due to enhanced vascular density or permeability.

In addition to satellite glia and endothelial cells, we also found that Jedi-1 is a novel marker for perineurial glia. Although the perineurium has not been shown to directly influence neuronal function, we know that it has important developmental roles in myelination and formation of the NMJ^11,40^. Additionally, it plays a crucial role in regeneration of peripheral nerves because it is one of the first cell types to bridge the nerve gap after injury and participates in phagocytosis of myelin and axonal debris during Wallerian degeneration^10^. After injury, the permeability of the blood nerve barrier breaks down to allow infiltration of peripheral immune cells that often contribute to neuropathic pain^41^. Our data show no statistically significant changes in Jedi-1 KO perineurium morphology or permeability under basal conditions; however, it is possible that loss of Jedi-1 from perineurial glia causes the release of soluble factors that alter neuronal activity in a paracrine signaling manner.

Our *in vitro* data demonstrating hypersensitivity of Jedi-1 KO DRG neurons to a depolarizing current stimulus was performed in mixed neuron-glia co-cultures that contain several cell types. Since these cultures were dissociated into single cells and the data was acquired under a constant flow of extracellular solution within one to two days after the dissection, it is unlikely that acute cell-cell contact or paracrine signaling are responsible for the change we observed *in vitro*. Rather, the mechanism is more likely to be developmental, since our mouse is a global (constitutive) knock-out of Jedi-1.

One of the interesting changes that we found in Jedi-1 KO DRG neurons was an increase in the number of cells that responded to capsaicin. Capsaicin is an agonist for the non-specific cation channel, TrpV1, which also responds to heat, acid, arachidonic acid derivatives (HETEs, AEA, etc.), mechanical, and osmotic pressure^42^. TrpV1 is expressed mostly in C fibers but also a subset of Aδ fibers^43^. A multitude of inflammatory chronic pain conditions including Irritable Bowel Syndrome, Rheumatoid Arthritis, and peripheral neuropathies have been shown to up-regulate TrpV1 activity through a number of different factors such as cytokines, prostaglandins, bradykinin, NGF, glutamine, and serotonin^44^. These molecules not only can be released by immune cells but also by satellite glial cells, Schwann cells, and endothelial cells when they become ‘activated’^45,46^. Since DRG neurons isolated from Jedi-1 KO mice are hyperactive and neuronal activity has also been shown to enhance TrpV1 activity, it is unclear whether TrpV1 underlies or is a by-product of neuronal activation. It is worth noting that 45% of Jedi-1 KO neurons responded to capsaicin but only ~30% of the KO cells were positive for the TrpV1 immunostaining. We suggest this is likely due to non-specific binding of capsaicin to other receptors and/or the relative insensitivity of antibody-based techniques.

Altered excitability of peripheral DRG neurons is associated with changes in nociception, raising the possibility that Jedi-1 KO mice and humans with loss-of-function (LOF) Jedi-1 SNPs such as rs12041331^22^ may have altered pain responses. Consistent with this possibility, our patch clamp electrophysiology experiments show that small diameter DRG neurons isolated from Jedi-1 KO animals exhibit enhanced excitability as demonstrated by the smaller rheobase and increase in the proportion of cells exhibiting a tonic rather than phasic firing pattern. Because our electrical recordings were from small diameter neurons, which are primarily nociceptors^47^, and TrpV1+ cells are also mostly small in diameter (Fig. 4E), and nociceptive^48^, we hypothesize that the cells patched for whole cell current clamp and the cells exhibiting enhanced capsaicin sensitivity during calcium imaging are most likely an overlapping population of pain-sensing cells. However, we did not evaluate TrpV1 expression or capsaicin sensitivity directly in the neurons we patched, therefore we cannot say for certain that these are the same cells.

A variety of voltage-gated ion channels and calcium-activated ion channels contribute to the electrical excitability of DRG neurons. Among these, voltage-gated sodium channels (VGSCs) play crucial roles in determining action potential firing patterns. Several VGSCs are highly expressed in small diameter nociceptive neurons including tetrodotoxin (TTX) sensitive channels (primarily Na_V_1.7 along with Na_V_1.3 and Na_V_1.6) and TTX resistant channels (Na_V_1.8, and Na_V_1.9). Human and mouse mutations in these sodium channel subunits lead to hereditary changes in human pain sensitivity^49^. This prompted us to investigate if loss of Jedi-1 affected sodium channel currents in small diameter DRG neurons. These whole cell voltage clamp experiments revealed changes to sodium channel function in neurons cultured from Jedi-1 KO mice, thereby corroborating the changes in neuronal excitability observed in current clamp recordings. Specifically, we found the overall current density (amount of current normalized for cell size) was not altered in the KO neurons compared to WT. However, we did find changes in the voltage-dependence of steady state inactivation which can serve as a biophysical fingerprint for the various sodium channel subtypes (Fig. 6C). The fractional contribution of the more hyperpolarized component was doubled in KO neurons (Table 2). Jedi-1 KO neurons also displayed a modest hyperpolarizing shift in activation, causing a larger and slightly hyperpolarized overlap of channel activation and inactivation, the so-called ‘window current’ (Fig. 6C). We speculate that the relative contribution of TTX-sensitive sodium channels (perhaps Na_V_1.7) is greater in the KO neurons. These channels inactivate in the hyperpolarized voltage range that is increased in the KO neurons and the modest hyperpolarizing shift in activation (Fig. 6B,C) is also consistent with an increased contribution from TTX-sensitive channels. Notably, the kinetics of closed-state inactivation are relatively slow for Na_V_1.7 channels^50,51^. This property means the channels can amplify subthreshold current inputs, thereby enhancing neuronal excitability^52^, consistent with the decrease in rheobase for KO neurons that we report (Fig. 5B). The shift from phasic to tonic evoked action potential firing in the KO neurons (Fig. 5E) might also involve altered sodium channel expression and/or function, although contributions from potassium, calcium, or chloride channels are also possible. Overall, our electrophysiology data identified altered sodium channel function in the KO neurons, but determining the precise mechanism(s) leading to increased excitability will require more extensive biophysical characterization.

In conclusion, we observed that Jedi-1 KO DRG neurons exhibited a change in neuronal excitability despite a lack of Jedi-1 expression in the neurons; rather, we found high Jedi-1 expression in perineurial glia and endothelial cells, and lower expression in a subset of satellite glia. We hypothesize that the change in neuronal activity is due to an indirect mechanism of intercellular interaction between these non-neuronal cells and the sensory neurons. It is interesting that all of the changes we found, capsaicin sensitivity, hyperactivity, and sodium current changes, are also found in inflammatory pain states. Our findings, like others, indicate that indirectly targeting neurons through glia may be an alternative treatment for chronic pain.

## Supplementary information

Supplementary information.

## Data Availability

The datasets generated during and/or analyzed during the current study are available from the corresponding author on reasonable request.

## References

[1] Lallemend, F. & Ernfors, P. Molecular interactions underlying the specification of sensory neurons. *Trends in Neurosciences*, 10.1016/j.tins.2012.03.006 (2012). 10.1016/j.tins.2012.03.006 22516617

[2] Oppenheim, R. Cell Death During Development Of The Nervous System. *Annu. Rev. Neurosci*., 10.1146/annurev.neuro.14.1.453 (1991). 10.1146/annurev.ne.14.030191.002321 2031577

[3] Wu, H. H. *et al*. Glial precursors clear sensory neuron corpses during development via Jedi-1, an engulfment receptor. *Nat. Neurosci*., 10.1038/nn.2446 (2009). 10.1038/nn.2446 PMC2834222 19915564

[4] Sullivan, C. S. *et al*. The adaptor protein GULP promotes Jedi-1-mediated phagocytosis through a clathrin-dependent mechanism. *Mol. Biol. Cell*., 10.1091/mbc.e13-11-0658 (2014). 10.1091/mbc.E13-11-0658 PMC4055271 24743597

[5] Scheib, J. L., Sullivan, C. S. & Carter, B. D. Jedi-1 and MEGF10 Signal Engulfment of Apoptotic Neurons through the Tyrosine Kinase Syk. *J. Neurosci*., 10.1523/jneurosci.6350-11.2012 (2012). 10.1523/JNEUROSCI.6350-11.2012 PMC3464495 22993420

[6] Hanani, M. Satellite glial cells in sensory ganglia: From form to function. *Brain Research Reviews*., 10.1016/j.brainresrev.2004.09.001 (2005). 10.1016/j.brainresrev.2004.09.001 15914252

[7] Huang, L. Y. M., Gu, Y. & Chen, Y. Communication between neuronal somata and satellite glial cells in sensory ganglia. *GLIA*., 10.1002/glia.22541 (2013). 10.1002/glia.22541 PMC3758405 23918214

[8] Kucenas, S. Perineurial glia. *Cold Spring Harb. Perspect. Biol*., 10.1101/cshperspect.a020511 (2015). 10.1101/cshperspect.a020511 PMC4448606 25818566

[9] Weerasuriya, A. & Mizisin, A. P. The Blood-Nerve Barrier: Structure and Functional Significance. In *The Blood-Brain and Other Neural Barriers* 149–173, 10.1007/978-1-60761-938-3_6 (2010). 10.1007/978-1-60761-938-3_6 21082370

[10] Lewis, G. M. & Kucenas, S. Perineurial Glia Are Essential for Motor Axon Regrowth following Nerve Injury. *J. Neurosci*., 10.1523/jneurosci.1906-14.2014 (2014). 10.1523/JNEUROSCI.1906-14.2014 PMC4166161 25232113

[11] Clark, J. K. *et al*. Mammalian Nkx2.2+ perineurial glia are essential for motor nerve development. *Dev. Dyn*., 10.1002/dvdy.24158 (2014). 10.1002/dvdy.24158 PMC4180512 24979729

[12] Weis, J., May, R. & Schröder, J. M. Fine structural and immunohistochemical identification of perineurial cells connecting proximal and distal stumps of transected peripheral nerves at early stages of regeneration in silicone tubes. *Acta Neuropathol*., 10.1007/BF00294509 (1994). 10.1007/BF00294509 7985496

[13] Fisch, A. S. *et al*. Genetic variation in the platelet endothelial aggregation receptor 1 gene results in endothelial dysfunction. *PLoS One*., 10.1371/journal.pone.0138795 (2015). 10.1371/journal.pone.0138795 PMC4583223 26406321

[14] Vandenbriele, C. *et al*. Platelet endothelial aggregation receptor-1: A novel modifier of neoangiogenesis. *Cardiovasc. Res*., 10.1093/cvr/cvv193 (2015). 10.1093/cvr/cvv193 26156496

[15] Kurtz, A. *et al*. The expression pattern of a novel gene encoding brain-fatty acid binding protein correlates with neuronal and glial cell development. *Development* (1994). 10.1242/dev.120.9.2637 7956838

[16] McDavid, S., Bauer, M. B., Brindley, R. L., Jewell, M. L. & Currie, K. P. M. Butanol isomers exert distinct effects on voltage-gated calcium channel currents and thus catecholamine secretion in adrenal chromaffin cells. *PLoS One*., 10.1371/journal.pone.0109203 (2014). 10.1371/journal.pone.0109203 PMC4183593 25275439

[17] Jewell, M. L., Breyer, R. M. & Currie, K. P. M. Regulation of calcium channels and exocytosis in mouse adrenal chromaffin cells by prostaglandin EP3 receptors. *Mol. Pharmacol*., 10.1124/mol.110.068569 (2011). 10.1124/mol.110.068569 PMC3102550 21383044

[18] Brindley, R. L., Bauer, M. B., Blakely, R. D. & Currie, K. P. M. An interplay between the serotonin transporter (SERT) and 5-HT receptors controls stimulus-secretion coupling in sympathoadrenal chromaffin cells. *Neuropharmacology*, 10.1016/j.neuropharm.2016.08.015 (2016). 10.1016/j.neuropharm.2016.08.015 PMC5028315 27544824

[19] Muona, P., Sollberg, S., Peltonen, J. & Uitto, J. Glucose transporters of rat peripheral nerve: Differential expression of GLUT1 gene by Schwann cells and perineurial cells *in vivo* and *in vitro*. *Diabetes*, 10.2337/diab.41.12.1587 (1992). 10.2337/diab.41.12.1587 1446800

[20] Pummi, K. P., Heape, A. M., Grénman, R. A., Peltonen, J. T. K. & Peltonen, S. A. Tight junction proteins ZO-1, occludin, and claudins in developing and adult human perineurium. *J. Histochem. Cytochem*., 10.1369/jhc.3A6217.2004 (2004). 10.1369/jhc.3A6217.2004 15258179

[21] Krivtsov, A. V. *et al*. Jedi - A novel transmembrane protein expressed in early hematopoietic cells. *J. Cell. Biochem*., 10.1002/jcb.21232 (2007). 10.1002/jcb.21232 17226770

[22] Izzi, B. *et al*. Allele-specific DNA methylation reinforces PEAR1 enhancer activity. *Blood*, 10.1182/blood-2015-11-682153 (2016). 10.1182/blood-2015-11-682153 27313330

[23] Weerasuriya, A. & Mizisin, A. P. The Blood-Nerve Barrier: Structure and Functional Significance. In, 10.1007/978-1-60761-938-3_6 (2010). 10.1007/978-1-60761-938-3_6 21082370

[24] White, F. A., Keller-Peck, C. R., Michael Knudson, C., Korsmeyer, S. J. & Snider, W. D. Widespread elimination of naturally occurring neuronal death in Bax- deficient mice. *J. Neurosci*. (1998). 10.1523/JNEUROSCI.18-04-01428.1998 PMC6792725 9454852

[25] Etchegaray, J. I. *et al*. Defective Phagocytic Corpse Processing Results in Neurodegeneration and Can Be Rescued by TORC1 Activation. *J. Neurosci*., 10.1523/jneurosci.1912-15.2016 (2016). 10.1523/JNEUROSCI.1912-15.2016 PMC4792933 26985028

[26] Marrone, M. C. *et al*. TRPV1 channels are critical brain inflammation detectors and neuropathic pain biomarkers in mice. *Nat. Commun*., 10.1038/ncomms15292 (2017). 10.1038/ncomms15292 PMC5436240 28489079

[27] Voolstra, O. & Huber, A. Post-Translational Modifications of TRP Channels. *Cells*, 10.3390/cells3020258 (2014). 10.3390/cells3020258 PMC4092855 24717323

[28] Dahlhamer, J. M. *et al*. Prevalence of chronic pain and high-impact chronic pain among adults — United States, 2016. *Morbidity and Mortality Weekly Report* (2018). 10.15585/mmwr.mm6736a2 PMC6146950 30212442

[29] Poulsen, J. N., Larsen, F., Duroux, M. & Gazerani, P. Primary culture of trigeminal satellite glial cells: A cell-based platform to study morphology and function of peripheral glia. *Int. J. Physiol. Pathophysiol. Pharmacol*. (2014). PMC3961097 24665354

[30] Takeda, M., Kitagawa, J., Takahashi, M. & Matsumoto, S. Activation of interleukin-1β receptor suppresses the voltage-gated potassium currents in the small-diameter trigeminal ganglion neurons following peripheral inflammation. *Pain*, 10.1016/j.pain.2008.06.015 (2008). 10.1016/j.pain.2008.06.015 18694623

[31] Dubový, P., Jančálek, R., Klusáková, I., Svíženská, I. & Pejchalová, K. Intra- and extraneuronal changes of immunofluorescence staining for TNF- and TNFR1 in the dorsal root ganglia of rat peripheral neuropathic pain models. *Cell. Mol. Neurobiol*., 10.1007/s10571-006-9006-3 (2006). 10.1007/s10571-006-9006-3 PMC11520733 16705482

[32] George, D., Ahrens, P. & Lambert, S. Satellite glial cells represent a population of developmentally arrested Schwann cells. *Glia*, 10.1002/glia.23320 (2018). 10.1002/glia.23320 29520852

[33] Lutz, A. B. *et al*. Schwann cells use TAM receptor-mediated phagocytosis in addition to autophagy to clear myelin in a mouse model of nerve injury. *Proc. Natl. Acad. Sci. USA*, 10.1073/pnas.1710566114 (2017). 10.1073/pnas.1710566114 PMC5617301 28874532

[34] Nanda, N. *et al*. Platelet endothelial aggregation receptor 1 (PEAR1), a novel epidermal growth factor repeat-containing transmembrane receptor, participates in platelet contact-induced activation. *J. Biol. Chem*., 10.1074/jbc.M413411200 (2005). 10.1074/jbc.M413411200 15851471

[35] Ohara, P. T. *et al*. Gliopathic pain: when satellite glial cells go bad. - PubMed - NCBI. (2009). 10.1177/1073858409336094 PMC2852320 19826169

[36] Hu, P., Bembrick, A. L., Keay, K. A. & McLachlan, E. M. Immune cell involvement in dorsal root ganglia and spinal cord after chronic constriction or transection of the rat sciatic nerve. *Brain. Behav. Immun*., 10.1016/j.bbi.2006.10.013 (2007). 10.1016/j.bbi.2006.10.013 17187959

[37] Kim, D., You, B., Lim, H. & Lee, S. J. Toll-like receptor 2 contributes to chemokine gene expression and macrophage infiltration in the dorsal root ganglia after peripheral nerve injury. *Mol. Pain*, 10.1186/1744-8069-7-74 (2011). 10.1186/1744-8069-7-74 PMC3192680 21951975

[38] Moreau, N. *et al*. Early alterations of Hedgehog signaling pathway in vascular endothelial cells after peripheral nerve injury elicit blood-nerve barrier disruption, nerve inflammation, and neuropathic pain development. *Pain*, 10.1097/j.pain.0000000000000444 (2016). 10.1097/j.pain.0000000000000444 26655733

[39] Beazley-Long, N. *et al*. VEGFR2 promotes central endothelial activation and the spread of pain in inflammatory arthritis. *Brain. Behav. Immun*., 10.1016/j.bbi.2018.03.012 (2018). 10.1016/j.bbi.2018.03.012 PMC6302073 29548992

[40] Kucenas, S. *et al*. CNS-derived glia ensheath peripheral nerves and mediate motor root development. *Nat. Neurosci*., 10.1038/nn2025 (2008). 10.1038/nn2025 PMC2657597 18176560

[41] Lim, T. K. Y. *et al*. Blood-nerve barrier dysfunction contributes to the generation of neuropathic pain and allows targeting of injured nerves for pain relief. *Pain*, 10.1016/j.pain.2014.01.026 (2014). 10.1016/j.pain.2014.01.026 24502843

[42] Rosenbaum, T. & Simon, S. TRPV1 Receptors and Signal Transduction. in *TRP Ion Channel Function in Sensory Transduction and Cellular Signaling Cascades* (eds. Liedtke, W. & Heller, S.), 10.1201/9781420005844.ch5 (2007). 21204486

[43] Guo, A., Vulchanova, L., Wang, J., Li, X. & Elde, R. Immunocytochemical localization of the vanilloid receptor 1 (VR1): relationship to neuropeptides, the P2X _3_ purinoceptor and IB4 binding sites. *Eur. J. Neurosci*., 10.1046/j.1460-9568.1999.00503.x (1999). 10.1046/j.1460-9568.1999.00503.x 10103088

[44] Brandt, M. R., Beyer, C. E. & Stahl, S. M. TRPV1 antagonists and chronic pain: Beyond thermal perception. *Pharmaceuticals*, 10.3390/ph5020114 (2012). 10.3390/ph5020114 PMC3763634 24288084

[45] Kevil, C. G. Endothelial cell activation in inflammation: Lessons from mutant mouse models. *Pathophysiology*, 10.1016/S0928468002000834 (2003). 10.1016/s0928468002000834 14567937

[46] Hunt, B. J. & Jurd, K. M. Endothelial cell activation. A central pathophysiological process. *BMJ* (1998). 10.1136/bmj.316.7141.1328 PMC1113063 9563977

[47] Julius, D. & Basbaum, A. Molecular mechanisms of nociception. *Nature*, 10.1038/35093019 (2001). 10.1038/35093019 11557989

[48] Caterina, M. J. *et al*. The capsaicin receptor: A heat-activated ion channel in the pain pathway. *Nature*, 10.1038/39807 (1997). 10.1038/39807 9349813

[49] Cummins, T. R., Sheets, P. L. & Waxman, S. G. The roles of sodium channels in nociception: Implications for mechanisms of pain. *Pain*, 10.1016/j.pain.2007.07.026 (2007). 10.1016/j.pain.2007.07.026 PMC2055547 17766042

[50] Cummins, T. R., Howe, J. R. & Waxman, S. G. Slow closed-state inactivation: A novel mechanism underlying ramp currents in cells expressing the hNE/PN1 sodium channel. *J. Neurosci*., 10.1523/jneurosci.18-23-09607.1998 (1998). 10.1523/JNEUROSCI.18-23-09607.1998 PMC6793269 9822722

[51] Herzog, R. I., Cummins, T. R., Ghassemi, F., Dib-Hajj, S. D. & Waxman, S. G. Distinct repriming and closed-state inactivation kinetics of Nav1.6 and Nav1.7 sodium channels in mouse spinal sensory neurons. *J. Physiol*., 10.1113/jphysiol.2003.047357 (2003). 10.1113/jphysiol.2003.047357 PMC2343279 12843211

[52] Rush, A. M., Cummins, T. R. & Waxman, S. G. Multiple sodium channels and their roles in electrogenesis within dorsal root ganglion neurons. *Journal of Physiology*, 10.1113/jphysiol.2006.121483 (2007). 10.1113/jphysiol.2006.121483 PMC2075388 17158175

