# Supplementary material for: Jedi-1 deficiency increases sensory neuron excitability through a non-cell autonomous mechanism
Source: Sci Rep. 2020 Jan 28;10:1300. doi: 10.1038/s41598-020-57971-2 (PMC6987110; doi:10.1038/s41598-020-57971-2)
Supplement: Supplementary file 1 — Supplementary information. [file 41598_2020_57971_MOESM1_ESM.pdf]

Supplementary information for

Jedi-1 deficiency increases sensory neuron excitability through a non-cell autonomous mechanism

Alexandra J. Trevisan[1], Mary Beth Bauer[2], Rebecca L. Brindley[3], Kevin P.M. Currie[3, #,*], and Bruce D. Carter[1,4,#,*]

[1] Department of Biochemistry, Vanderbilt University School of Medicine, Nashville, TN, USA
[2] Anesthesiology, Vanderbilt University School of Medicine, Nashville, TN, USA
[3] Department of Biomedical Sciences, Cooper Medical School of Rowan University, Camden, NJ, USA
[4] Vanderbilt Brain Institute, Vanderbilt University School of Medicine, Nashville, TN, USA

[#] These authors contributed equally to this work

* To whom correspondence should be addressed: Bruce D. Carter, Department of Biochemistry, 625 Light Hall, Vanderbilt University School of Medicine, Nashville, TN 37232; Email: bruce.carter@Vanderbilt.edu; Kevin P.M. Currie, Department of Biomedical Sciences, Cooper Medical School of Rowan University, Camden, NJ, USA; Email: currie@rowan.edu.

Supplemental Figure S1: Mouse model validation.

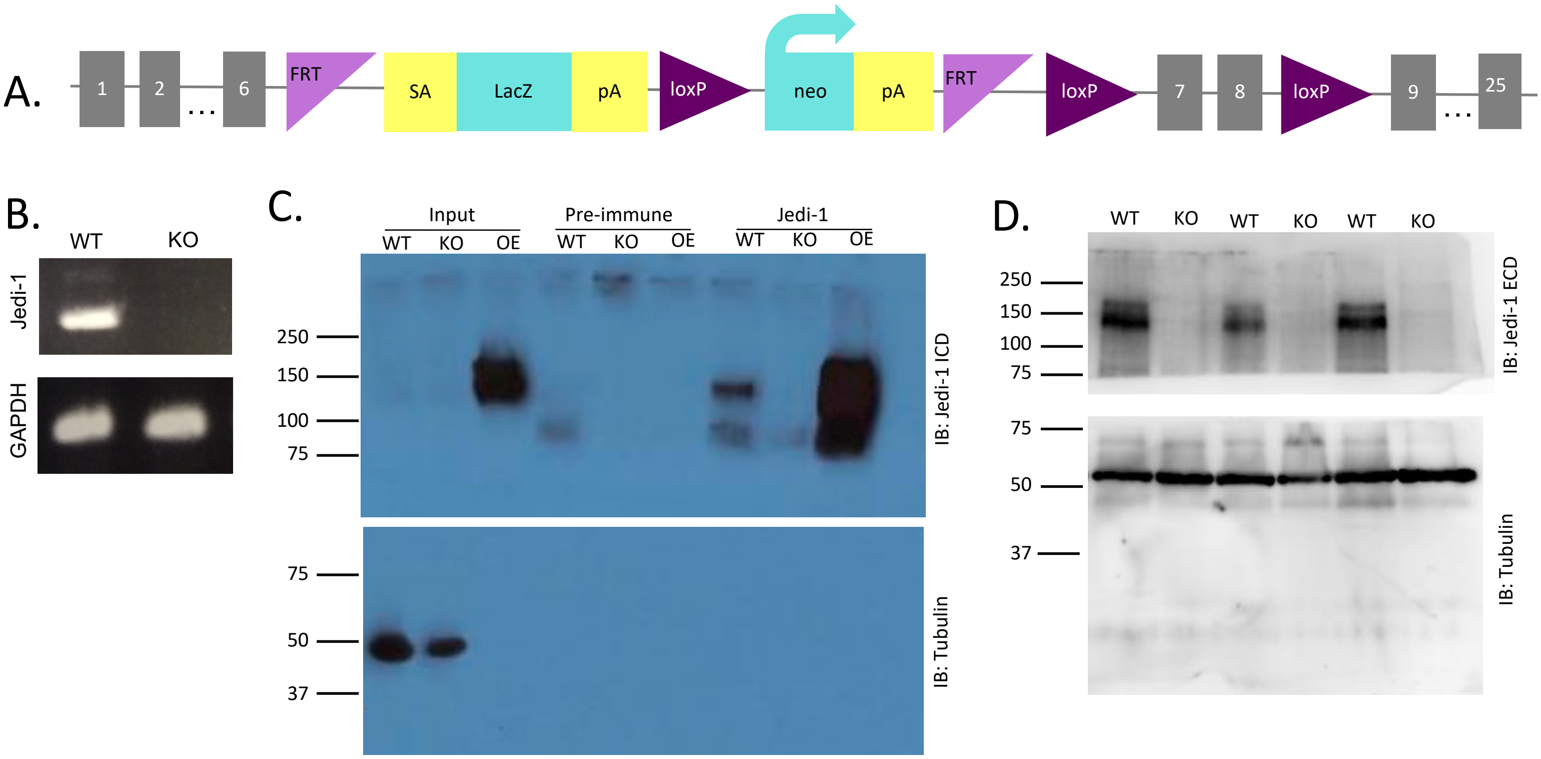

Supplemental Figure S1: Mouse model validation. (A) Scheme of KOMP Jedi-1 KO mouse model. SA = splice acceptor, pA = poly A tail. Exons numbered in grey boxes (not drawn to scale). (B) RT-PCR of WT and KO brain lysates using primers for Jedi-1 that span the KOMP lacZ insertion. Control primers for GAPDH used. (C) IP-western using two different antibodies generated against the intracellular domain (ICD) of Jedi-1. Samples are adult whole brain lysates from WT or KO animals or HeLa cells overexpressing (OE) Jedi-1. Markers in kDa. The full length of the gels are shown. (D) Western blot using a polyclonal antibody generated against the entire extracellular domain (ECD) of Jedi-1 using pooled DRGs from 3 replicate WT and KO adult mice. Markers in kDa. The full length of the gels are shown.

Supplemental Figure S2: Jedi-1 is a novel marker for perineurial glia.

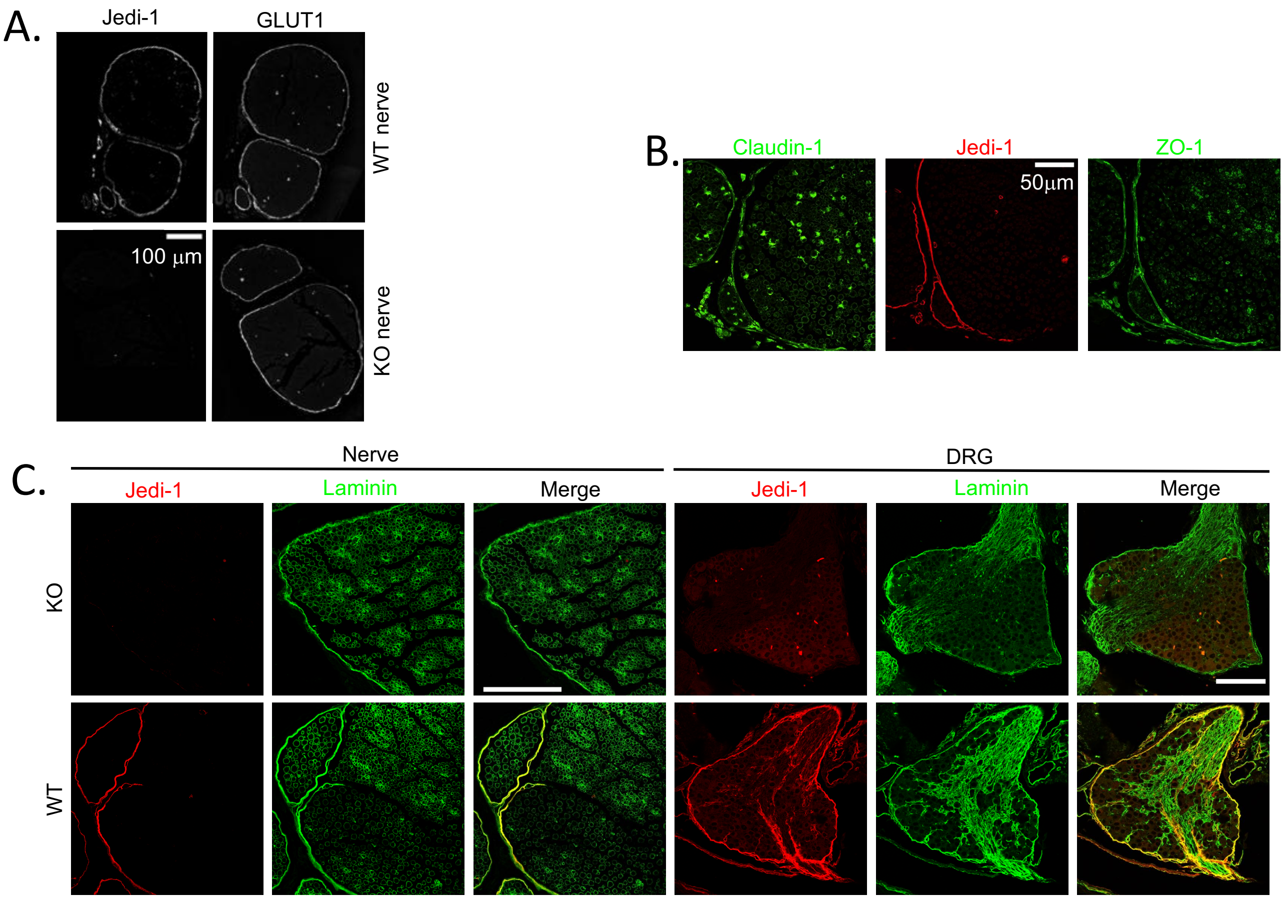

Supplemental Figure S2: Jedi-1 is a novel marker for perineurial glia. (A) Consecutive sciatic nerve cross sections stained for Jedi-1 (left) and GLUT1 (right). These antibodies cannot be co-stained together, so consecutive sections were stained and rotated to show the nerve in the same orientation on a black background. Both Jedi-1 and GLUT1 are expressed in perineurial glia and endothelial cells. (B) WT sciatic nerve was serially sectioned and consecutive cross sections were stained for Claudin-1 (green), Jedi-1 (red), and ZO-1 (green). Antibodies are not compatible for co-staining. (C) WT and KO nerve cross sections or DRGs were co-stained for Jedi-1 (cyan) and laminin (magenta). White shows co-localization.

Supplemental Figure S3: Peripheral glia are not altered in the absence of Jedi-1.

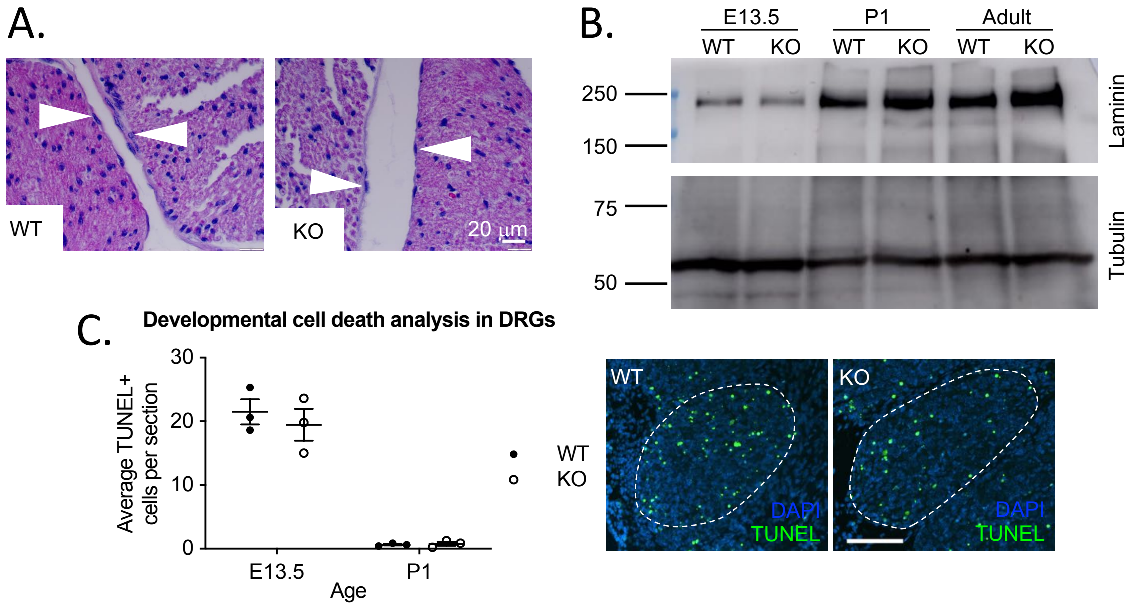

Supplemental Figure S3: Peripheral glia are not altered in the absence of Jedi-1. (A) H&E of WT and KO adult sciatic nerves. Arrows point to the perineurial cell layer. (B) Western blot from DRG lysates collected at various developmental ages in WT and KO mice immunoblotted with a pan-laminin antibody and tubulin as a loading control. (C) Left shows the quantification of TUNEL staining at E13.5 or P1 in WT or KO mice. Serial sections were taken at 5 microns through all spinal levels of ganglia and every 12th section stained. Average number of TUNEL+ cells per section per ganglia were averaged for each animal and 3 animals analyzed per genotype and time point. Error bars represent SEM. No statistically significant differences between genotypes. Right shows representative picture of TUNEL staining (green) and DAPI (blue) of E13.5 DRGs, outlined in white dotted lines. Scale bar is 100 microns.
